# Association between lipid-lowering drugs and allergic diseases: A Mendelian randomization study^☆^

**DOI:** 10.1016/j.waojou.2024.100899

**Published:** 2024-04-10

**Authors:** Yinsong Xu, Yuanzhi Li

**Affiliations:** a Clinical Medical College, Chengdu University of Traditional Chinese Medicine, Chengdu, 610075, Sichuan, China; b Ya'an Polytechnic College, Ya'an, 625000, Sichuan, China; c Department of Anorectal Surgery, Shenzhen TCM Anorectal Hospital (Futian), Shenzhen, 518000, China; d Department of Traditional Chinese Medicine, The Afliated Hospital of Southwest Medical University, Luzhou, 646000, China

**Keywords:** Lipid-lowering drugs, Allergic diseases, Mendelian randomization

## Abstract

**Background:**

Several observational studies suggest a possible link between lipid-lowering drugs and allergic diseases. However, inferring causality from these studies can be challenging due to issues such as bias, reverse causation, and residual confounding. To investigate the potential causal effect of lipid-lowering drugs, proprotein convertase subtilisin/kexin type 9 (PCSK9) inhibitors and 3-hydroxy-3-methyl-glutaryl-coenzyme A reductase (HMGCR) inhibitors, on allergic diseases (allergic asthma, allergic conjunctivitis, atopic dermatitis, allergic rhinitis, and allergic urticaria), we performed a Mendelian randomization (MR)-based study.

**Methods:**

We employed MR and summary-data-based MR (SMR), analyzing genome-wide association study (GWAS) data from people of European descent. Single nucleotide polymorphisms (SNPs) were employed as instrumental variables. We selected 2 types of genetic measures to represent the impact of lipid-lowering drugs, including genetic variants near or within drug target genes correlated with low-density lipoprotein cholesterol (LDL-C), and expression quantitative trait loci of drug target genes. The inverse-variance weighted (IVW)-MR approach was the primary utilized MR method, while sensitivity analyses were used to test the robustness of the results. We used SMR analysis as a supplementary analytical method, applying the heterogeneity in dependent instruments (HEIDI) test to assess if the observed correlation between gene expression and outcome was due to a linkage situation.

**Results:**

The IVW-MR analysis revealed significant evidence for an association between PCSK9-mediated LDL-C reduction and a decrease in the risk of allergic asthma (odds ratio [OR] = 1.31, 95% confidence interval [CI] = 1.11–1.56; P < 0.01). Likewise, SMR analysis discovered an augmented expression of PCSK9 being linked with a heightened susceptibility to allergic asthma (OR = 1.21, 95% CI = 1.03–1.43; P = 0.02). No consistent evidence was found for other associations in either analysis.

**Conclusion:**

Our findings support a potential causal relationship between PCSK9 activity and an increased risk of allergic asthma. Thus, PCSK9 inhibitors, which reduce PCSK9 activity, might be considered a priority in future clinical trials investigating drugs for allergic asthma prevention or treatment.

Allergic diseases, such as allergic asthma (AA) and atopic dermatitis (AD), are hypersensitivity reactions generated by specific immune cells or antibodies and constitute significant and prevalent global health concerns.^1^, ^2^, ^3^ These diseases not only affect the respiratory and digestive systems but can also impact the skin and other organs. Frequently coexisting, these disorders substantially reduce the quality of life for those affected.^4^, ^5^, ^6^ Driven by societal, environmental, and lifestyle changes, the prevalence and morbidity of allergic diseases continue to rise, positioning them as a prominent health issue.^7^^,^^8^ Therefore, understanding the relationship between allergic diseases and other risk factors is crucial for developing effective disease prevention and treatment strategies to minimize their societal burden and impact.

Serum lipids, including cholesterol, triglycerides (TG), low-density lipoprotein-cholesterol (LDL-C), and high-density lipoprotein-cholesterol (HDL-C),^9^ which play an essential role in maintaining cellular structure, energy metabolism, signal transduction, and material transport.^10^ Accumulating evidence has unveiled a substantial association between dyslipidemia and allergic diseases. For example, it has been shown that high levels of TG and LDL-C and low levels of HDL-C are associated with asthma, and AD is associated with lower levels of TG and LDL.^11^, ^12^, ^13^ Considering these findings, the relationship between lipid-lowering drugs and allergic diseases has received considerable attention. Commonly prescribed lipid-lowering drugs, such as 3-hydroxy-3-methyl-glutaryl-coenzyme A reductase (HMGCR) inhibitors and proprotein convertase subtilisin/kexin type 9 (PCSK9) inhibitors, play pivotal roles in managing dyslipidemia and reducing cardiovascular risk. HMGCR inhibitors, also known as statins, are the most widely used lipid-lowering drugs; they can inhibit the HMGCR enzyme, thereby reducing cholesterol and LDL-C levels by inhibiting intracellular synthesis of cholesterol.^14^ PCSK9 inhibitors, relatively new targeted therapeutic modalities for lowering LDL-C, can reduce the level of LDL-C in plasma by inhibiting PCSK9 and preventing it from binding to the low-density lipoprotein receptor (LDLR), which in turn prevents LDLR degradation and facilitates LDL-C clearance.^15^ Recent studies have found that these drugs also possess pleiotropic effects, such as anti-inflammatory and anti-allergic abilities, beyond lipid control.^16^, ^17^, ^18^

Nonetheless, clinical and experimental data concerning the anti-allergic effects of lipid-lowering drugs are inconclusive and contradictory, leaving the validity of these effects open to debate. For instance, the effect of statins on asthma remains disputed; a double-blind, randomized crossover study found no evidence of simvastatin having anti-inflammatory properties in patients with asthma, while it had significant effects on Th17-mediated neutrophilic inflammation and airway hyperreactivity in a mouse model of severe asthma.^19^^,^^20^ Therefore, it is uncertain if a causal link exists between lipid-lowering drugs and allergic diseases. The inconsistency can be attributed to human observational research's susceptibility to reverse causality and confounding factors. Thus, well-designed studies are needed to reach a definitive conclusion.

Mendelian randomization (MR) offers a new approach to statistical analysis, assessing the influence of modifiable exposures on outcomes using genetic variants as instrumental variables (IVs).^21^ Since the distribution of genetic variants is randomized at conception, potential biases due to confounding variables and reverse causality that can distort the exposure-outcome association can be mitigated through MR analysis, hence yielding more robust outcomes.^22^ Numerous fields have already employed MR studies.^23^, ^24^, ^25^, ^26^

Therefore, we performed a two-sample MR analysis to investigate the potential association between lipid-lowering drugs and allergic diseases.

## Data and methods

### Study design

**Fig. 1:**
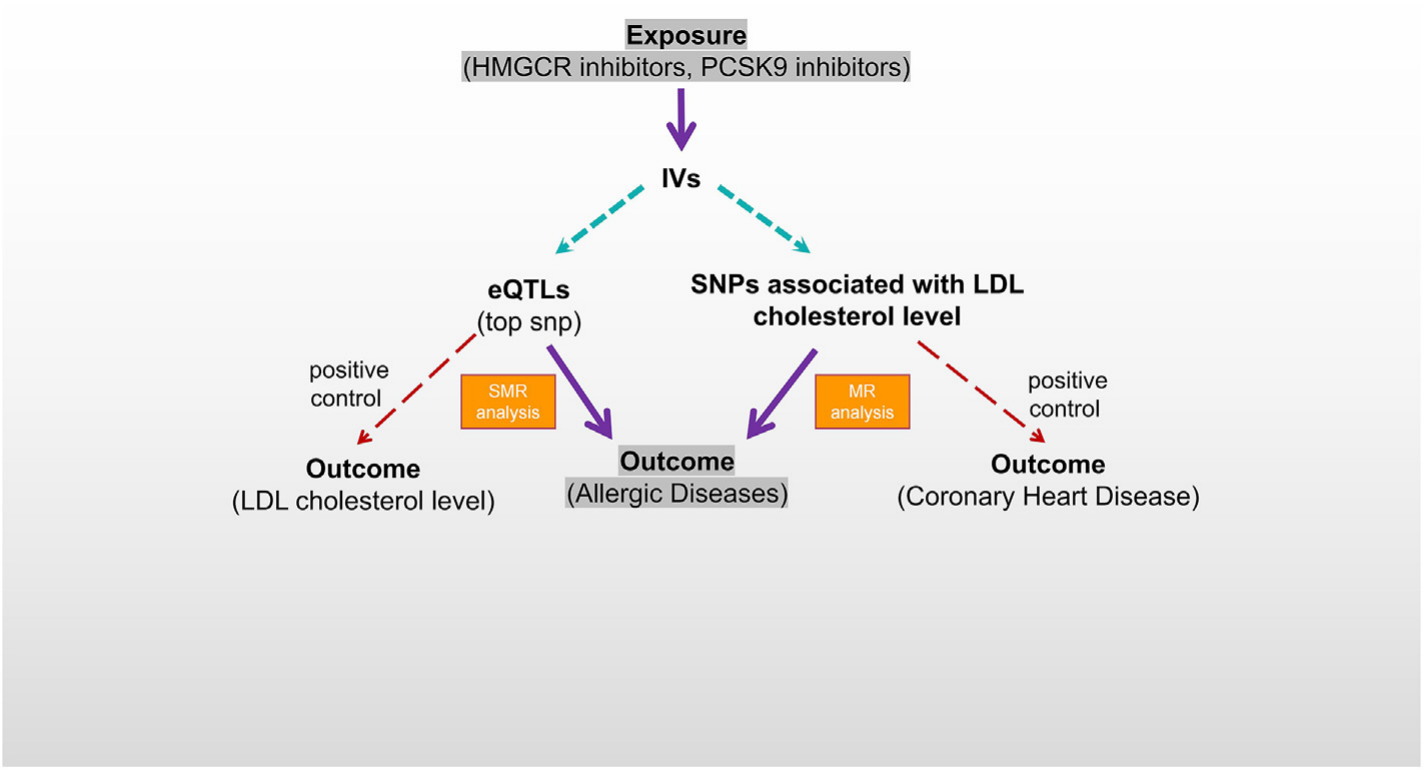
Overview of the design

We employed 2 analytical approaches to examine the effect of lipid-lowering drugs on the risk of allergic diseases. Our primary method was a two-sample MR analysis, supplemented by summary-data-based MR (SMR) analysis to reinforce our methodological strategy. A degree of consistency in phenotypic findings between the primary and supplementary analyses was interpreted as an indication of a credible causal correlation. Food and Drug Administration-approved lipid-lowering drugs, including HMGCR inhibitors and PCSK9 inhibitors were included as exposures in the study. Due to the lack of significant expression quantitative trait loci (eQTLs) for Niemann-Pick C1-like 1 (NPC1L1) in the blood, NPC1L1 inhibitors were excluded. Five common allergic diseases, including AA, allergic conjunctivitis (AC), AD, allergic rhinitis (AR), and allergic urticaria (AU) were designated as outcomes in our study. Fig. 1 details the overall design of the study.

For the MR methodology, we adopted low-density lipoprotein-cholesterol, a well-established outcome of lipid-lowering treatment, as the biomarker. Genetic variations, particularly *cis*-variants located within genes that encode protein targets of lipid-lowering drugs, were identified from a Genome-Wide Association Study (GWAS) dataset for LDL-C. These variants served as proxies for the use of lipid-lowering medication and were subsequently used to examine the association with allergic diseases. Additionally, we examined the relationship between these exposures and coronary heart disease (CHD), with CHD serving as a positive control given the frequent prescription of lipid-lowering drugs for this condition.

In the SMR methodology, eQTLs corresponding to HMGCR and PCSK9 genes were selected to serve as proxies for exposure to each class of lipid-lowering medication. As a positive control, we also examined the relationship between these exposures and the levels of LDL-C.

To ensure the accuracy of the results, 3 key assumptions needed to be confirmed throughout the entire process: (1) relevance assumption: the selected single nucleotide polymorphisms (SNPs) must be robustly associated with the target proteins; (2) exclusivity assumption: the selected SNPs cannot be directly related to allergic diseases; (3) independence assumption: the selected SNPs are independent of any potential confounding factors.

Given that this study represents a reevaluation of previously collected and published data, no additional ethical approval was needed.

### Data sources

We utilized summary statistics derived from GWAS databases. Our exposure and outcome data were sourced from 2 separate, non-overlapping populations of European descent to circumvent potential pleiotropic biases that could emerge in cross-lineage situations.^21^ The LDL-C GWAS summary data were retrieved from the UK Biobank, incorporating a sample size of 440,546. The GWAS summary data for allergic diseases and CHD were collected from the FinnGen biobank, encompassing diverse sample sizes: 219,753 for AA, 377,277 for AC, 350,062 for AD, 370,158 for AR, 366,969 for AU, and 377,277 for CHD. The eQTLs summary-level data were obtained either from the eQTLGen Consortium^27^ (https://www.eqtlgen.org/) or GTEx Consortium V8 (https://gtexportal.org/). Details on the data sources are provided in Supplementary Table 1.

### Selection of genetic instruments

When using genetic variants associated with LDL-C levels as instrumental variables, we selected SNPs located in or near (±100 kb) each drug's target gene. Specifically, we chose those SNPs that showed a substantial association with LDL-C levels at a genome-wide significance level (*P* < 5.0 × 10^−8^). Our investigation was confined to common SNPs with a minor allele frequency (MAF) exceeding 1%, to ensure substantial population coverage. To enhance the effectiveness of the IVs, we allowed SNPs to exhibit weak linkage disequilibrium (LD) amongst themselves, characterized by an r^2^ value below 0.30.^28^

We assessed the strength of the IVs using the F-statistic, an indicator of the proportion of exposure variance accounted for by the IVs.^29^ The F-statistic was calculated using the formula: F = [R^2^/(1-R^2^)]ⅹ[(N–K-1)/K],^30^ where R^2^ represents the extent of exposure variance, N stands for the total number of samples, and K represents the number of SNPs in the GWAS. An F-statistic exceeding 10 was deemed indicative of a sufficiently robust instrument, thereby minimizing the potential for weak bias of IVs^31^ in the subsequent MR analysis. We discarded any SNPs that demonstrated an association with allergic diseases and exhibited a *P*-value less than 5ⅹ10^−8^ from the IVs prior to conducting the MR analysis. In instances where certain SNPs were absent from the outcome GWAS datasets, we substituted them with corresponding overlapping proxy SNPs (LDr^2^ > 0.8). Then, through harmonization procedures, we eliminated palindromic SNPs with an intermediate allele frequency (MAF >0.3) and any incompatible SNPs.

When using eQTLs as instruments, we selected common eQTL SNPs (MAF >1%) that were significantly (*P* < 5.0 × 10^−8^) associated with the expression of HMGCR or PCSK9 in blood samples. This study included only *cis*-eQTLs, which are defined as eQTLs located within 1 megabase (Mb) on either side of the gene they encode.^28^

### Statistical analysis

To investigate the causal impact of lipid-lowering drugs on allergic diseases, we used genetic variants associated with LDL-C as instruments and applied 5 regression models: inverse variance weighted (IVW), simple mode, weighted mode, weighted median, and MR Egger. The IVW method, our primary approach for MR analysis, integrated the Wald ratio of each SNP on the outcome to generate a pooled causal estimate. The random effects IVW represented the benchmark for MR results,^26^ with other methods providing supplementary support. The results are presented as odds ratios (OR) with their corresponding 95% confidence intervals (CI).

Furthermore, to validate the reliability of our MR estimates, we performed sensitivity analyses that included Cochran's Q test to evaluate heterogeneity and the *P*-value from the MR-Egger regression intercept to investigate potential pleiotropy. Additionally, we carried out leave-one-out sensitivity tests and used the MR-pleiotropy residual sum and outlier (MR-PRESSO) method to address any potentially pleiotropic SNPs. A *P*-value <0.05 was used as the threshold for statistical significance. Data analysis was conducted using R (version 4.2.2), the “TwoSample MR” and “MR-PRESSO” packages.

In our study, we used an approach which integrates GWAS and eQTL data to investigate the relationship between gene expression and the outcomes of interest.^32^ To generate effect estimates, the SMR method was employed.

Additionally, we used the heterogeneity in dependent instruments (HEIDI) test to differentiate pleiotropy from linkage. A HEIDI test with a P-value <0.01 suggests that the observed association may be due to linkage rather than pleiotropy.^33^^,^^34^ The aforementioned data analysis was conducted using SMR software (version 1.3.1).

## Results

### Selection of genetic instruments

For the MR method, we selected 19 SNPs from the HMGCR and 33 SNPs from the PCSK9 genes to serve as proxies for HMGCR and PCSK9 inhibitors. Each SNP selected met both the independence and relevance assumptions. For the SMR method, rs6453133 and rs472495 were selected as top SNPs^35^ to represent HMGCR and PCSK9 inhibitors, respectively. Additionally, we calculated the F-statistics for each SNP to exclude weak IVs (F < 10) and found no weak IVs. Detailed information about these SNPs is available in Supplementary Table 2.

### Statistical analysis

**Fig. 2:**
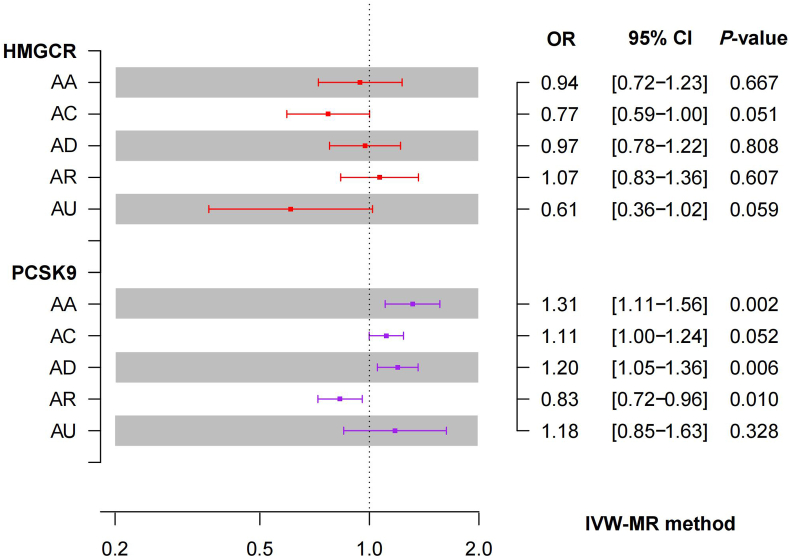
IVW-MR analysis of the association between LDL-C levels mediated by HMGCR and PCSK9 genes and Allergic Diseases

The IVW-MR analysis provided suggestive evidence for the association between PCSK9-mediated LDL-C reduction and risk of AA (odds ratio [OR] = 1.31, 95% confidence interval [CI] = 1.11–1.56; *P* < 0.01), AD (OR = 1.20, 95% CI = 1.05–1.36; *P* < 0.01), and AR (OR = 0.83, 95% CI = 0.72–0.96; *P* = 0.01). These results indicate that PCSK9 inhibitors may have a protective role against AA and AD, but a potential detrimental effect against AR. However, the relationship between HMGCR-mediated LDL-C reduction and allergic diseases remained unclear as the analysis did not provide substantiating evidence. Primary results are illustrated in Fig. 2.

**Fig. 3:**
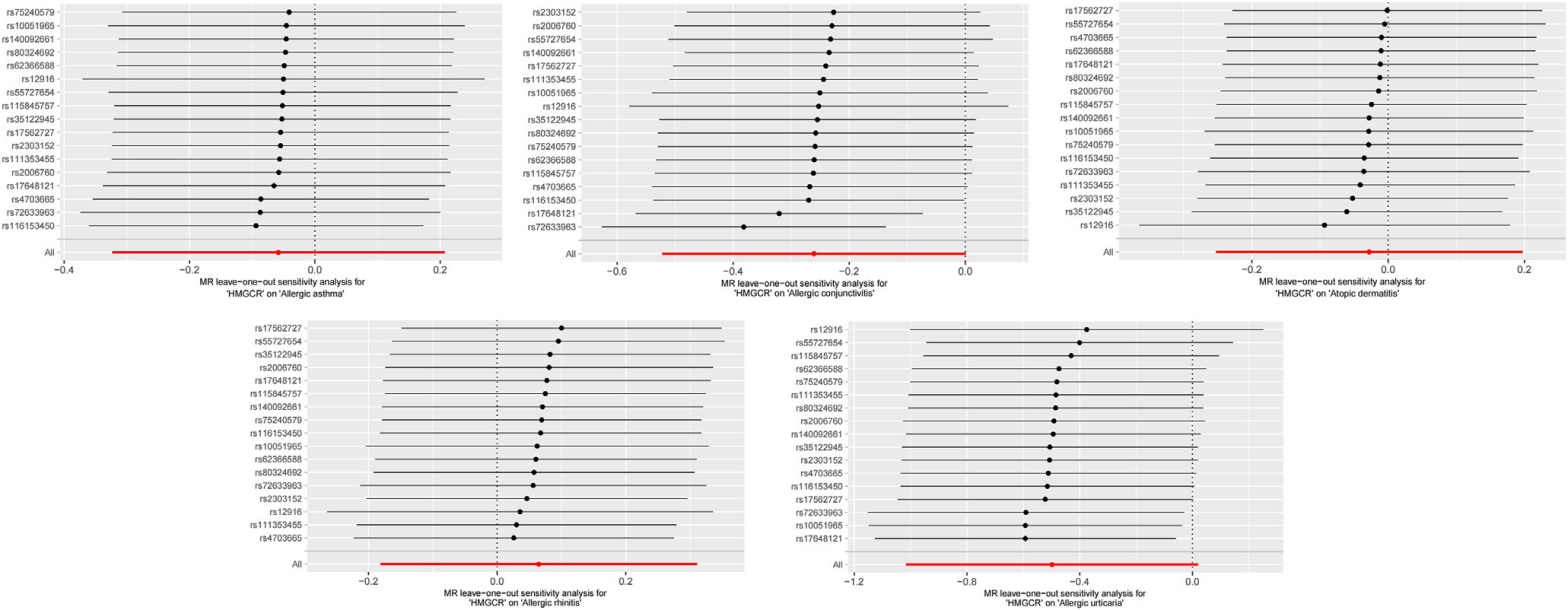
MR leave−one−out sensitivity analysis for HMGCR genes on Allergic Diseases

**Fig. 4:**
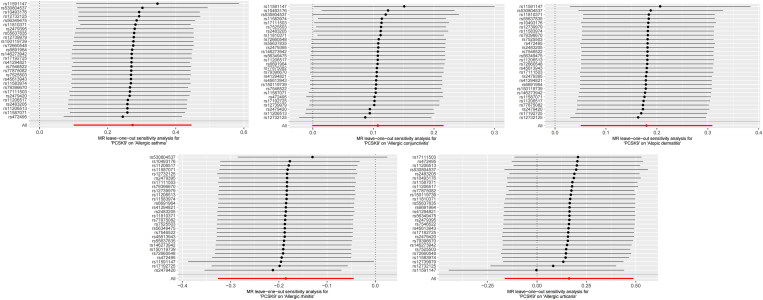
MR leave−one−out sensitivity analysis for PCSK9 genes on Allergic Diseases

No horizontal pleiotropy was observed in our MR analysis. The observed heterogeneity in the relationship between HMGCR-mediated LDL-C and the risk of AC was noteworthy (IVW Q statistics = 33.44; Qdf = 16; Qpval <0.01), but did not invalidate the MR estimates. Furthermore, no significant differences were observed in the estimated causal effects when we removed individual SNPs and repeated the MR analysis in a leave-one-out analysis. Details are available in Fig. 3 and Fig. 4. These results indicated robust findings that were not influenced by the exclusion of single IVs. The MR-PRESSO did not identify any outlier SNPs, confirming no deviation in the estimates.

**Fig. 5:**
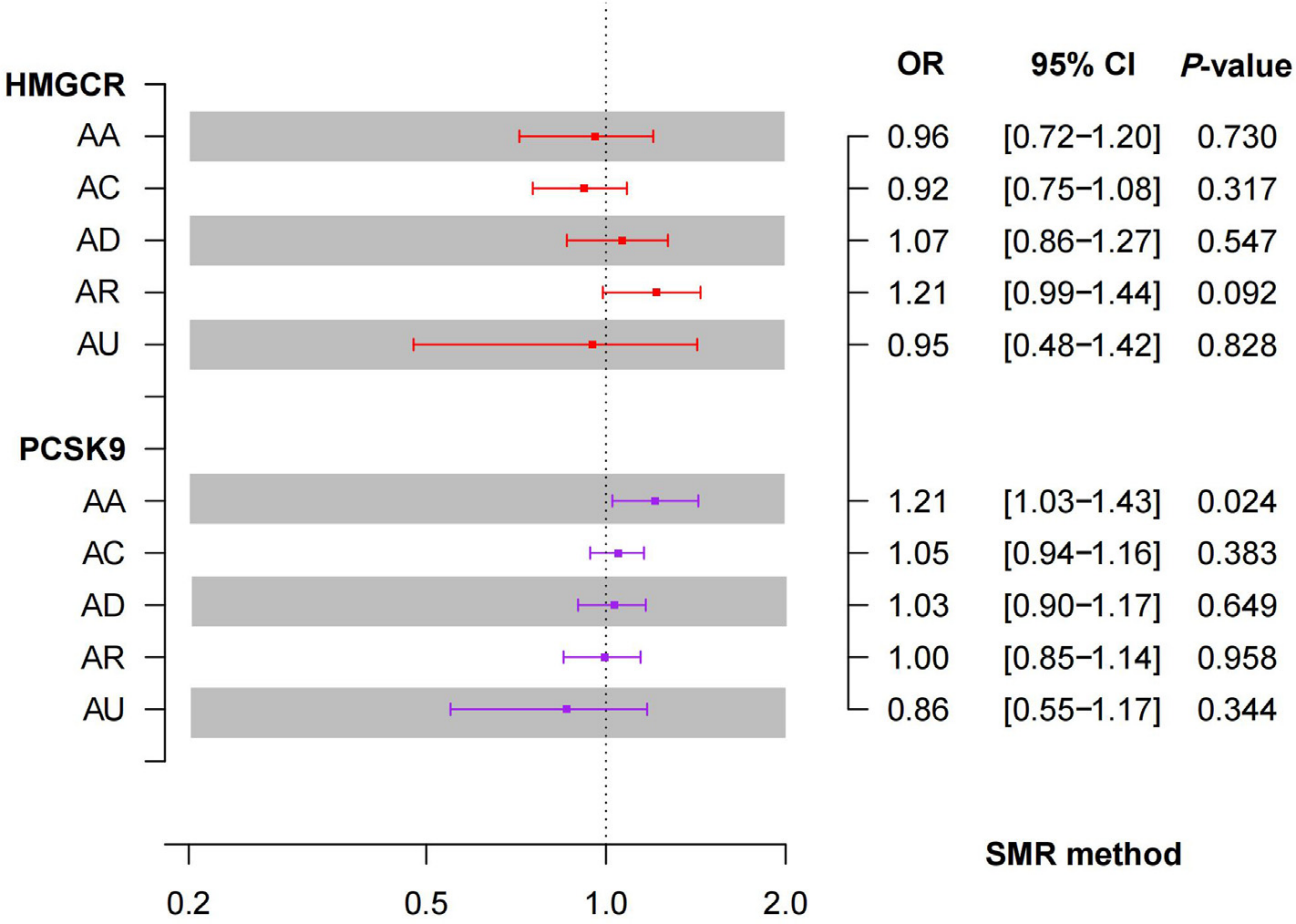
SMR analysis of the association between HMGCR and PCSK9 Gene Expression and Allergic Diseases

Additionally, the SMR analysis demonstrated an association between an augmented expression of the PCSK9 gene in the blood and an elevated risk of AA (OR = 1.21, 95% CI = 1.03−1.43; *P* = 0.02). According to the HEIDI test, the observed association was not the result of a linkage (*P* = 0.81), implying a potential protective role of PCSK9 inhibitors against AA. Despite these findings, the correlation between the expression of HMGCR and allergic diseases remains uncertain, as this research was unable to demonstrate a significant association. The primary results are depicted in Fig. 5.

Detailed results are available in Supplementary Table 3.

## Discussion

Using the two-sample MR method, we investigated the potential causal relationship between lipid-lowering drugs and allergic diseases. The integration of results from both MR and SMR methods supported a potential protective effect of PCSK9 inhibitors against AA. Although our study also detected associations between PCSK9 inhibitors and other allergic diseases (a protective role in AD, but a harmful effect on AR), these were not consistently reflected across both methods. These disparities indicate the need for further investigation to accurately determine the relationships between PCSK9 inhibitors and various allergic conditions. No significant correlations were found between HMGCR inhibitors and allergic diseases, aligning with previous MR research that explored the overall effects of statins, reporting no change in risk for asthma or AR.^36^ While our study suggests a potential protective role of PCSK9 inhibitors against AA, it is noteworthy to consider contrasting findings reported by Xie et al. Their Mendelian randomization study indicates a causal relationship between PCSK9 inhibitors and an increased risk of asthma.^37^ The discrepancy in results could stem from several factors, including genetic instruments utilized and outcome definitions. It is well known that asthma comprises multiple phenotypes, including allergic and nonallergic asthma, childhood and adult asthma, and obesity-related manifestations, with differences in presentation, pathogenesis, and course of the disease among different subtypes.^38^ Additionally, variations in the pleiotropic effects of PCSK9 inhibitors across different disease contexts may contribute to divergent findings. Further research exploring these potential mechanisms and conducting meta-analyses to synthesize evidence from multiple studies could provide a more comprehensive understanding of the complex relationship between PCSK9 inhibitors and asthma risk.

PCSK9 inhibitors, known to effectively and securely diminish the levels of LDL-C, reduce the activity of PCSK9 and slow down the breakdown of LDLR, often used to treat CHD. These drugs have been spotlighted for their potential additional advantages, such as anti-inflammatory properties, in recent research.^39^ Numerous studies have confirmed a correlation between PCSK9 and inflammation. For example, 1 study assessing the impact of PCSK9 suppression and overexpression on physiological parameters in mice revealed that high levels of PCSK9 led to increased systemic release of the inflammatory interleukin (IL)-6 and exacerbated lung inflammation. Conversely, PCSK9 deficiency led to reduced circulating levels of IL-6 and lessened organ inflammation.^40^ A similar pattern was reported in another animal study, where overexpression of PCSK9 in macrophages led to an increased release of pro-inflammatory cytokines triggered by oxLDL. Conversely, silencing PCSK9 lowered the expression of inflammation markers such as tumor necrosis factor (TNF)-α, IL-1β, monocyte chemoattractant protein-1, toll-like receptor 4, and nuclear factor kappa B (NF-κB).^41^ Thus, the expression of PCSK9 might contribute to inflammation, and PCSK9 inhibitors might reduce inflammation by downregulating the expression of PCSK9.

Inflammation plays a key-role in asthma, primarily characterized by chronic airway inflammation. Through comprehensive and in-depth studies in recent years, asthma has been classified into “Type-2-high” and “Type-2-low” asthma by different molecular mechanisms.^42^ “Type-2-high” asthma, including AA,^43^ often involved an atopic response with Th2-predominant inflammation (associated with the release of IL-4, IL-5, and IL-13)^44^^,^^45^ and marked by elevated levels of inflammation markers such as C-reactive protein, IL-6, and total immunoglobin E (IgE).^46^ Studies has demonstrated that both Th2 cytokines and proinflammatory factors are important in the inflammatory characteristics of AA.^47^^,^^48^ IL-4 can induce allergen-specific B lymphocytes to produce IgE;^49^ IL-5 can evoke eosinophilic inflammation by recruiting eosinophils;^50^ IL-4 and IL-13 can induce mucus hypersecretion and goblet cell metaplasia,^51^ their collaborative action with IL-5 fosters airway hyperreactivity,^52^ while their synergistic effect with transforming growth factor (TGF)-β and IL-6 facilitates airway remodeling.^53^^,^^54^ Differential additive effects of these factors on the airways contribute to the pathogenesis of AA.^55^ NF-κB functions as a principal orchestrator of gene transcription, primarily associated with immune and inflammatory activities^56^，it governs the expression of more than 500 genes.^57^ In allergic asthma, NF-κB can promote the expression of inflammatory factors it regulates, such as IL-4 and IL-5.^58^ In addition, NF-κB activation is strongly associated with airway remodeling.^59^ The NF-κB signaling disorders are closely related to the pathophysiology of many diseases, including AA,^60^ it is widely recognized as a typical proinflammatory pathway for the production of proinflammatory cytokines such as IL-4, IL-5, IL-13, IL-1, and TNF-α.^61^ Meanwhile, studies have revealed that PCSK9 could promote the inflammatory factors by activating the NF-κB pathway.^62^ Therefore, it is reasonably assumed that the protective effect of PCSK9 inhibitors against AA could be largely attributed to their inhibition of the NF-κB pathway.

Combined with our findings, considering the established safety profile and clear mechanism of action of these approved pharmacological agents,^63^ and their repurposing potential which would expedite the development of novel drugs against AA, PCSK9 inhibitors could be strong candidates for future clinical trials aiming to uncover new therapeutic approaches for this pathology.

Nonetheless, it is important to acknowledge the limitations of our study. First, the GWAS datasets used were solely from European populations, which means the applicability of our results to other populations remains uncertain. Further research involving different ethnic groups is needed to confirm the generalizability of our results. Second, our outcome data did not provide a detailed description of the severity of allergic diseases. Therefore, further studies are required to determine the link between the use of lipid-lowering drugs and varying severity level of allergic diseases. Third, genetically predicted drug effects may differ from those observed during clinical trials. Hence, future research is essential to validate this causality, which is essential for generating practical treatment recommendations. Fourth, it is important to acknowledge the potential variation in the utilization of lipid-lowering drugs across different regions and healthcare systems. While our study provides valuable insights into the potential causal relationship between lipid-lowering drugs and allergic diseases, it is essential to consider that the prescription patterns and availability of these medications may differ, particularly in developing countries where they might not be utilized as first-line treatments. Therefore, future research exploring the effects of lipid-lowering drugs on allergic diseases should take into account such regional disparities in drug usage and access to healthcare. Furthermore, the association between dyslipidemia and obesity adds another layer of complexity to our findings. Asthma patients with obesity may have influenced the observed association between lipid-lowering drugs and the risk of asthma. Therefore, while our results suggest a potential link between lipid-lowering drugs and asthma risk, particularly in individuals with obesity, caution is warranted in attributing these findings solely to allergic pathology. These limitations highlight the need for further research to better characterize the subtypes of asthma and elucidate the underlying mechanisms driving the observed associations. Future studies incorporating more refined phenotypic data and considering the complex interplay between dyslipidemia, obesity, and asthma subtypes will contribute to a more comprehensive understanding of the relationship between lipid-lowering drugs and allergic diseases.

## Conclusion

In summary, the current research provides MR evidence supporting PCSK9 inhibitors as potential therapeutic agents for treating AA. Nonetheless, considering the complex pharmacological mechanisms of these medications, clinical trials are crucial to validate these findings. Future research should aim at uncovering the underlying mechanisms that govern the relationship between PCSK9 inhibitors and AA, thereby paving the way for the development of effective treatment strategies.

## Abbreviations

AA, Allergic asthma; AC, Allergic conjunctivitis; AD, Atopic dermatitis; AR, Allergic rhinitis; AU, Allergic urticaria; CHD, Coronary heart disease; CI, Confidence intervals; eQTL, Expression quantitative trait loci; GWAS, Genome-Wide Association Study; HDL-C, High-density lipoprotein-cholesterol; HEIDI, Heterogeneity in dependent instruments; HMGCR, 3-hydroxy-3-methyl-glutaryl-coenzyme A reductase; IgE, Immunoglobin E; IL, Inflammatory interleukin; IV, Instrumental variable; IVW, Inverse variance weighted; LD, Linkage disequilibrium; LDL-C, Low-density lipoprotein-cholesterol; LDLR, Low-density lipoprotein receptor; MAF, Minor allele frequency; Mb, Megabase; MR, Mendelian randomization; MR-PRESSO, MR-pleiotropy residual sum and outlier; NF-κB, Nuclear factor kappa B; NPC1L1, Niemann-Pick C1-like 1; OR, Odds ratios; PCSK9, Proprotein convertase subtilisin/kexin type 9; SMR, Summary-data-based MR; SNP, Single nucleotide polymorphism; TG, Triglycerides; TGF, Transforming growth factor; TNF, Tumor necrosis factor.

## Funding

The current study was supported by Scientifific Research Project of Science and Technology Department of Sichuan Province (No.2022YFS0408).

## Availability of data and materials

All data are incorporated into the article and its supplementary material.

## Authors' contributions

Yinsong Xu: analyzed the data, and wrote the manuscript; Yuanzhi Li: designed the study, critically read and edited the manuscript. All authors read and approved the final manuscript.

## Ethics approval

Given that this study represents a reevaluation of previously collected and published data, no additional ethical approval was needed.

## Authors’ consent for publication

All authors have approved the manuscript and agree with its submission to World Allergy Organization Journal.

## Declaration of competing interest

The authors declare that they have no known competing financial interests or personal relationships that could have appeared to influence the work reported in this paper.

## References

[1] Wang Y., Liu T., Wan Z. (2023). Investigating causal relationships between the gut microbiota and allergic diseases: a mendelian randomization study. Front Genet.

[2] Khasawneh R., Al-Hiary M., Al-Abadi B. (2019). Total and specific immunoglobulin E for detection of most prevalent aeroallergens in a Jordanian cohort. Med Arch.

[3] Li Y., Hu H., Zhang T. (2021). Increase in indoor inhalant allergen sensitivity during the COVID-19 pandemic in south China: a cross-sectional study from 2017 to 2020. J Asthma Allergy.

[4] Kilanowski A., Thiering E., Wang G. (2023). Allergic disease trajectories up to adolescence: characteristics, early-life, and genetic determinants. Allergy.

[5] Blöndal V., Sundbom F., Zhou X. (2023). Allergic sensitisation and type-2 inflammation is associated with new-onset and persistent allergic disease. Clin Transl Allergy.

[6] Vos T., Lim S.S., Abbafati C. (2020). Global burden of 369 diseases and injuries in 204 countries and territories, 1990–2019: a systematic analysis for the Global Burden of Disease Study 2019. Lancet.

[7] Agache I., Laculiceanu A., Spanu D. (2023). The concept of one health for allergic diseases and asthma. Allergy, Asthma & Immunology Research.

[8] Donald K., Finlay B.B. (2023). Early-life interactions between the microbiota and immune system: impact on immune system development and atopic disease. Nat Rev Immunol.

[9] Rosen E.M., Kotlarz N., Knappe D.R.U. (2022). Drinking water–associated PFAS and fluoroethers and lipid outcomes in the GenX exposure study. Environ Health Perspect.

[10] Wang B., Wei J., Huangfu Q. (2022). Identification of resolvin D1 and protectin D1 as potential therapeutic agents for treating kidney stones. Oxid Med Cell Longev.

[11] Gao S., Zeng Q., Zeng Y. (2022). High density lipoprotein inhibited group II innate lymphoid cells proliferation and function in allergic rhinitis. Allergy Asthma Clin Immunol : Official Journal of the Canadian Society of Allergy and Clinical Immunology.

[12] Su X., Ren Y., Li M. (2018). Association between lipid profile and the prevalence of asthma: a meta-analysis and systemic review. Curr Med Res Opin.

[13] Lim J.E., Kim H.M., Kim J.H. (2023). Association between dyslipidemia and asthma in children: a systematic review and multicenter cohort study using a common data model. Clinical and Experimental Pediatrics.

[14] Uemura N., Hayashi H., Baba H. (2022). Statin as a therapeutic agent in gastroenterological cancer. World J Gastrointest Oncol.

[15] Schonck W.A.M., Stroes E.S.G., Hovingh G.K. (2024). Long-Term efficacy and tolerability of PCSK9 targeted therapy: a review of the literature. Drugs.

[16] Sharpton S.R., Loomba R. (2023). Emerging role of statin therapy in the prevention and management of cirrhosis, portal hypertension, and HCC. Hepatology.

[17] Fu C.-H., Tsai W.-C., Lee T.-J. (2016). Simvastatin inhibits IL-5-induced chemotaxis and CCR3 expression of HL-60-derived and human primary eosinophils. PLoS One.

[18] Tang Z., Jiang L., Peng J. (2012). PCSK9 siRNA suppresses the inflammatory response induced by oxLDL through inhibition of NF-κB activation in THP-1-derived macrophages. Int J Mol Med.

[19] Menzies D., Nair A., Meldrum K.T. (2007). Simvastatin does not exhibit therapeutic anti-inflammatory effects in asthma. J Allergy Clin Immunol.

[20] Chen Y.-R., Xiang X.-D., Sun F. (2023). Simvastatin reduces NETosis to attenuate severe asthma by inhibiting PAD4 expression. Oxid Med Cell Longev.

[21] Xiang M., Wang Y., Gao Z. (2022). Exploring causal correlations between inflammatory cytokines and systemic lupus erythematosus: a Mendelian randomization. Front Immunol.

[22] Su Z., Wu Z., Liang X. (2023). Diabetic retinopathy risk in patients with unhealthy lifestyle: a Mendelian randomization study. Front Endocrinol.

[23] Ahn K., Penn R.B., Rattan S. (2023). Mendelian randomization analysis reveals a complex genetic interplay among atopic dermatitis, asthma, and gastroesophageal reflux disease. Am J Respir Crit Care Med.

[24] Li R., Chen Y., Zhao A. (2022). Exploring genetic association of insomnia with allergic disease and asthma: a bidirectional Mendelian randomization study. Respir Res.

[25] Brito Nunes C., Huang P., Wang G. (2023). Mendelian randomization study of maternal coffee consumption and its influence on birthweight, stillbirth, miscarriage, gestational age and pre-term birth. Int J Epidemiol.

[26] Shi H., Zhao H., Zhang W. (2023). COVID-19 is not a causal risk for miscarriage: evidence from a Mendelian randomization study. J Assist Reprod Genet.

[27] Võsa U., Claringbould A., Westra H.-J. (2021). Large-scale cis- and trans-eQTL analyses identify thousands of genetic loci and polygenic scores that regulate blood gene expression. Nat Genet.

[28] Huang W., Xiao J., Ji J. (2021). Association of lipid-lowering drugs with COVID-19 outcomes from a Mendelian randomization study. Elife.

[29] Feng Y., Liu X., Tan H. (2022). Causal association of peripheral immune cell counts and atrial fibrillation: a Mendelian randomization study. Frontiers in Cardiovascular Medicine.

[30] Yang M., Wan X., Zheng H. (2023). No evidence of a genetic causal relationship between ankylosing spondylitis and gut microbiota: a two-sample mendelian randomization study. Nutrients.

[31] Burgess S., Thompson S.G. (2011). CRP CHD Genetics Collaboration. Avoiding bias from weak instruments in Mendelian randomization studies. Int J Epidemiol.

[32] Zhu Z., Zhang F., Hu H. (2016). Integration of summary data from GWAS and eQTL studies predicts complex trait gene targets. Nat Genet.

[33] Wu Y., Zeng J., Zhang F. (2018). Integrative analysis of omics summary data reveals putative mechanisms underlying complex traits. Nat Commun.

[34] Lu A.T., Hannon E., Levine M.E. (2017). Genetic architecture of epigenetic and neuronal ageing rates in human brain regions. Nat Commun.

[35] Tian R., Pan Y., Etheridge T.H.A. (2020). Pitfalls in single clone CRISPR-cas9 mutagenesis to fine-map regulatory intervals. Genes.

[36] Yang G., Schooling C.M. (2021). Investigating genetically mimicked effects of statins via HMGCR inhibition on immune-related diseases in men and women using Mendelian randomization. Sci Rep.

[37] Xie W., Li J., Du H. (2023). Causal relationship between PCSK9 inhibitor and autoimmune diseases: a drug target Mendelian randomization study. Arthritis Res Ther.

[38] Messelodi D., Giuliani C., Cipriani F. (2022). C5 and SRGAP3 polymorphisms are linked to paediatric allergic asthma in the Italian population. Genes.

[39] Chen H., Chen X. (2023). PCSK9 inhibitors for acute coronary syndrome: the era of early implementation. Frontiers in Cardiovascular Medicine.

[40] Dwivedi D.J., Grin P.M., Khan M. (2016). Differential expression of PCSK9 modulates infection, inflammation, and coagulation in a murine model of sepsis. Shock.

[41] Tang Z.-H., Peng J., Ren Z. (2017). New role of PCSK9 in atherosclerotic inflammation promotion involving the TLR4/NF-κB pathway. Atherosclerosis.

[42] (2019). The cytokines of asthma. Immunity.

[43] Curto E., Mateus-Medina É.F., Crespo-Lessmann A. (2022). Identification of two eosinophil subsets in induced sputum from patients with allergic asthma according to CD15 and CD66b expression. Int J Environ Res Publ Health.

[44] Wang K., Wang L., Zhao G. (2023). Mechanistic study of salidroside on ovalbumin-induced asthmatic model mice based on untargeted metabolomics analysis. Food Funct.

[45] Grunstein M.M. (2023). Homeostatic glucocorticoid signaling in airway smooth muscle: a roadmap to asthma pathogenesis. Front Endocrinol.

[46] Shateri Z., Hosseini S.A., Abolnezhadian F. (2022). Pomegranate extract supplementation improves lung function parameters and IL-35 expression in participants with mild and moderate persistent allergic asthma: a randomized, double-blind, placebo-controlled trial. Front Nutr.

[47] Yu T., Yu Y., Ma Y. (2023). Inhibition of CREB promotes glucocorticoids action on airway inflammation in pediatric asthma by promoting ferroptosis of eosinophils. Allergol Immunopathol.

[48] Hynes G.M., Hinks T.S.C. (2020). The role of interleukin-17 in asthma: a protective response?. ERJ Open Research.

[49] Hsieh C.-C., Ng Y.-Y., Li W.-S. (2022). Ameliorative effect of imperatorin on dermatophagoides pteronyssinus-induced allergic asthma by suppressing the Th2 response in mice. Molecules.

[50] Lloyd C.M., Snelgrove R.J. (2018). Type 2 immunity: expanding our view. Science Immunology.

[51] Singh S., Dutta J., Ray A. (2023). Airway epithelium: a neglected but crucial cell type in asthma pathobiology. Diagnostics.

[52] Rosskopf S., Jahn-Schmid B., Schmetterer K.G. (2018). PD-1 has a unique capacity to inhibit allergen-specific human CD4+ T cell responses. Sci Rep.

[53] Li C., Lu Y., Du S. (2017). Dioscin exerts protective effects against crystalline silica-induced pulmonary fibrosis in mice. Theranostics.

[54] Chen Z., Bai F.-F., Han L. (2018). Targeting neutrophils in severe asthma via siglec-9. Int Arch Allergy Immunol.

[55] Gandhi N.A., Pirozzi G., Graham N.M.H. (2017). Commonality of the IL-4/IL-13 pathway in atopic diseases. Expet Rev Clin Immunol.

[56] Napetschnig J., Wu H. (2013). Molecular basis of NF-κB signaling. Annu Rev Biophys.

[57] Rocha D.M., Caldas A.P., Oliveira L.L. (2016). Saturated fatty acids trigger TLR4-mediated inflammatory response. Atherosclerosis.

[58] Xu R., Deng H., Gan L. (2022). Chinese herbal component, Praeruptorin E, enhances anti-asthma efficacy and prevents toxicity of aminophylline by targeting the NF-κB/PXR/CYP3A4 pathway. Ann Transl Med.

[59] Xian Z., Choi Y.H., Zheng M. (2020). Imperatorin alleviates ROS-mediated airway remodeling by targeting the Nrf2/HO-1 signaling pathway. Biosci, Biotechnol, Biochem.

[60] Li J., Zheng M., Wang C. (2020). Cryptotanshinone attenuates allergic airway inflammation through negative regulation of NF-κB and p38 MAPK. Biosci, Biotechnol, Biochem.

[61] Chu H., Jiang S., Liu Q. (2018). Sirtuin1 protects against systemic sclerosis–related pulmonary fibrosis by decreasing proinflammatory and profibrotic processes. Am J Respir Cell Mol Biol.

[62] Cai J., Jiang Y., Chen F. (2023). PCSK9 promotes T helper 1 and T helper 17 cell differentiation by activating the nuclear factor-κB pathway in ankylosing spondylitis. Immunity, Inflammation and Disease.

[63] Stefanik M., Valdes J.J., Ezebuo F.C. (2020). FDA-approved drugs efavirenz, tipranavir, and dasabuvir inhibit replication of multiple flaviviruses in vero cells. Microorganisms.

